# The Foreign Journals: Midwifery

**Published:** 1840-04

**Authors:** 


### MIDWIFERY.

### *On the Influence of Digitalis on the Contractions of the Uterus.* By M. Piedagnel.

The object of this paper is to recommend, from theory and from a limited practice, the administration of digitalis in false labour pains. The cases in which it has been found most useful are those in which during gestation there are vague pains in the uterus producing considerable suffering and fatigue, and those in which after delivery the pains continue for an unusually long period, for example, for more than two or three hours after the expulsion of the placenta. In these and in certain other cases in which it has been a common custom to administer opium, the author suggests that digitalis should be employed, which he believes acts by diminishing the force and frequency of the contractions of the uterus, as it is well known to do those of the heart. He has generally used an infusion of one fresh leaf or of two dried leaves in a cup of water, which is taken at once, or a drink, containing from thirty to sixty centigrammes of powdered leaves, of which the patient takes a spoonful every half hour or hour till the pains cease.

*Bulletin Général de Thérapeutique. Janvier,* 1840.

